# Monoclonal gammopathy of renal significance from the perspective of nephrologists

**DOI:** 10.1007/s44313-024-00027-5

**Published:** 2024-08-12

**Authors:** Kootae Park, Soon Hyo Kwon

**Affiliations:** Division of Nephrology, Hyonam Kidney Laboratory, Soonchunhyang University Hospital, 59 Daesagwan-Ro, Yongsan-Gu, Seoul, South Korea

**Keywords:** Chronic kidney disease, Kidney biopsy, Light chain, Monoclonal gammopathy

## Abstract

Kidney disease is a frequent complication of multiple myeloma and other malignancies associated with monoclonal gammopathies. Additionally, dysproteinemia-related kidney disease can occur independently of overt multiple myeloma or hematologic malignancies. Monoclonal gammopathy of renal significance (MGRS) is a spectrum of disorders in which a monoclonal immunoglobulin produced by a benign or premalignant B-cell or plasma cell clone causes kidney damage. MGRS-associated renal disease manifests in various forms, including immunoglobulin-associated amyloidosis, monoclonal immunoglobulin deposition diseases (light chain, heavy chain, and combined light and heavy chain deposition diseases), proliferative glomerulonephritis with monoclonal immunoglobulin deposits, C3 glomerulopathy with monoclonal gammopathy, and light chain proximal tubulopathy. Although MGRS is a nonmalignant or premalignant hematologic condition, it has significant renal implications that often lead to progressive kidney damage and, eventually, end-stage kidney disease. This review discusses the epidemiology, pathogenesis, and management of MGRS and focuses on the perspective of nephrologists.

## Introduction

Hematologic disorders, including multiple myeloma, which present with monoclonal gammopathies (MGs), are often associated with kidney disease [1]. MG is the presence of a monoclonal immunoglobulin produced by an abnormal B-cell clone in serum, urine, or both. The clone usually consists of plasma cells when the monoclonal immunoglobulin is immunoglobulin G, immunoglobulin A, immunoglobulin D, or light chain only; however, it consists of lymphoplasmacytes when the monoclonal immunoglobulin is immunoglobulin M [2]. MGs are particularly common among older individuals [3]. Secreted monoclonal immunoglobulins are often implicated in organ damage, with the kidneys being the most common target [4]. The results of kidney dysfunction evaluations can be used to diagnose hematologic disorders such as multiple myeloma and light chain disease [5].

Because the number of pathologic renal conditions associated with various hematologic disorders has increased, in 2012, the International Kidney and Monoclonal Gammopathy Research Group introduced MG of renal significance (MGRS) as a new diagnosis that comprises a group of diseases in which a monoclonal immunoglobulin is found in the blood and affects kidney function [5]. MGRS comprises nonmalignant hematologic conditions that produce monoclonal immunoglobulins associated with kidney disease [4]. By definition, these disorders do not meet the diagnostic criteria for overt or symptomatic multiple myeloma or lymphoproliferative disorders. MGRS, which causes kidney damage, is distinguished by MG of undetermined significance (MGUS) without evidence of end-organ damage. With MGRS, kidney disease manifests as glomerular disease, tubulopathy, and vascular involvement. MGRS is also classified as organized or nonorganized deposits [6]. Organized deposits can be subdivided into fibrillar, microtubular, inclusion, or crystallin categories. Monoclonal immunoglobulin deposition disease and proliferative glomerulonephritis with monoclonal immunoglobulin deposits (PGNMID) involve nonorganized monoclonal immunoglobulin deposits. However, some MGRS do not involve deposits and are classified as miscellaneous.

The prevalence of MGRS increases with age [4]. Chronic kidney disease (CKD) is becoming more common among older individuals [7]. MGRS may be underdiagnosed in older individuals because of the presence of other comorbid conditions that can mask MGRS-related signs and symptoms. Many developed and developing countries' populations are rapidly aging [8]; therefore, the awareness of MGRS should be increased among nephrologists, hematologists, and pathologists. Early detection of MGRS is crucial for preventing severe clinical outcomes. Therefore, we described the diagnosis and management of MGRS, focusing on the perspective of nephrologists [4].

### Pathogenesis and characteristics of MGRS

#### How does dysproteinemia cause kidney injury in MGRS?

Dysproteinemia is the presence of monoclonal immunoglobulins in the blood [9]. High tumor burden-induced kidney injury, known as light chain cast nephropathy, is characterized by monoclonal light chain binding to the Tamm-Horsfall protein [10]. This process requires increased serum free light chain levels in patients with conditions such as multiple myeloma, lymphoplasmacytic lymphoma, and high-grade chronic lymphocytic leukemia [11]. However, the tumor burden and quantity of monoclonal immunoglobulins associated with MGRS are not high.

Previous studies have suggested several hypotheses regarding the mechanisms by which dysproteinemia causes kidney damage [12]. Immunoglobulins' potential toxicity may stem from their ability to bind to other proteins, a characteristic determined by their physicochemical properties, which are influenced by their amino acid sequences [12]. Immunoglobulins are secreted into the bloodstream and subsequently filtered at the glomerulus. Because the kidney receives 20% of the cardiac output, clonal disorders that lead to kidney injury are unsurprising [12]. Light and heavy chains are low-molecular-weight proteins that are relatively freely filtered at the glomerulus and subsequently reabsorbed and hydrolyzed by the proximal tubules via endocytosis by the megalin/cubulin receptor system [13]. Although immunoglobulins primarily interact with cells in the glomerulus, monoclonal light chains and heavy chains can affect the functions of various cells across the entire nephron [9]. Free light chains isolated from patients with plasma cell dyscrasia and kidney injury are particularly prone to self-associate and form higher-molecular-weight aggregates under physiological conditions than those of patients with plasma cell dyscrasia but no kidney injury [14]. Mice injected with free light chains from patients experienced kidney injury, indicating that specific clones of free light chains lead to kidney injury [15]. These findings suggest that the primary molecular structure is an important determinant of the injury pattern. The deposited light and heavy chains interact with resident glomerular cells, activating inflammation and tissue injury [16]. This mechanism may explain the renal damage observed in patients with amyloidosis and monoclonal immunoglobulin deposition diseases. Misfolding of an immunoglobulin fragment leads to the formation of toxic amyloid multimers and amyloid fibrils [17]. Finally, these products are secreted extracellularly and deposited with other components such as amyloid fibrils. Increased levels of matrix metalloproteinases also destroy the mesangial matrix [18].

The mechanisms of injury associated with PGNMID, immunotactoid glomerulonephritis, fibrillary glomerulonephritis, membranoproliferative glomerulonephritis with monoclonal immunoglobulins, and cryoglobulinemic glomerulonephritis remain poorly understood [19]. C3 glomerulopathy is characterized by dominant C3 deposits with a membranoproliferative pattern. In C3 glomerulopathy, the monoclonal immunoglobulin overactivates the alternative complement pathway, acting as an autoantibody against factor H or as a stabilizer of C3 convertase [20, 21]. This results in glomerular C3 deposits without pathologic immunoglobulin deposition. A similar mechanism may be involved in the association between MG and thrombotic microangiopathy [22].

#### MGRS is not a benign kidney condition

In terms of outcomes, reduced kidney function in patients with MGRS frequently progresses to end-stage renal disease (ESRD). One study showed that seven (22%) of 32 patients with biopsy-proven MGRS experienced progression to ESRD during follow-up (median, 30.3 months) [23]. Additionally, 12 (37.5%) patients had persistent renal dysfunction, and one (3.1%) patient had persistent microhematuria with normal creatinine levels and no proteinuria. Among patients with ESRD development, three received no treatment. These findings suggest that treatment that targets the underlying MG improves renal outcomes.

The effects of different renal lesion subtypes associated with MGRS on the renal prognosis remain unclear. However, a multicenter, retrospective study found differences between the disease severity and survival rates of amyloidosis-associated MGRS (MGRS-A) and nonamyloidosis-associated MGRS (MGRS-NA) [24]. Kidney disease was more severe in the MGRS-NA group, and a higher proportion of patients had an increased creatinine level and low glomerular filtration rate and required dialysis at presentation. Among patients who received treatment, the renal response (> 30% reduction of proteinuria, absence of > 25% reduction of the estimated glomerular filtration rate) was less common than the hematologic response. Patients with MGRS-A had a lower likelihood of a renal response than those with MGRS-NA. Overall, 25% of patients with a relatively good hematologic response (complete remission or excellent partial response) did not experience a renal response. No difference in hematologic responses was found between the two groups [24]. However, the subtypes of renal lesions associated with MGRS and renal survival (defined as dialysis initiation) did not differ. Another study showed no difference in the renal prognoses associated with MGRS-A and MGRS-NA [25]. Furthermore, the degree of tubular atrophy did not affect renal survival during a follow-up period of 24 months [26]. However, the short follow-up periods and small cohort sizes of these studies may have limited the ability to draw definitive conclusions regarding the relationship between the histological subtypes of MGRS and renal survival [26].

A recent study compared the kidney outcomes of patients with CKD with either monoclonal MGUS or MGRS and those of patients without MG [27]. Univariate analysis showed that patients with MGRS were at higher risk for ESRD than those without MG, but patients with MGUS were not. However, a multivariable analysis found that, after adjusting for traditional risk factors, the risk of progression to ESRD in the MGRS group and the CKD without MG group did not differ, suggesting that the higher proteinuria level and lower estimated glomerular filtration rate of MGRS at presentation affect renal outcomes. Notably, treating all patients with MGRS likely impacted the kidney outcomes observed during this study [27]. Additionally, systemic treatment improved the kidney outcomes of patients with C3 glomerulopathy with MG [20]. Patients who achieved a hematologic response after chemotherapy that targeted the B-cell clone had higher renal response rates and median renal survival rates than those who received conservative/immunosuppressive therapy for C3 glomerulopathy with MG. Chemotherapy also improved the renal prognosis of patients with MGRS with immunoglobulin M MG [25]. One study found that chemotherapy led to the discontinuation of dialysis (2 months) [26]. Kidney function with MGRS can deteriorate despite treatment; however, appropriate treatment may improve kidney outcomes.

#### Clinicopathologic characteristics of MGRS

The incidence and prevalence of MGRS remain unclear. However, the estimated incidence of MGUS is 7 to 59 times higher than that of glomerular disease. According to case series reports, the median age of patients with MGRS is between the late 50s and early 60s [28, 29]. Furthermore, because of their age, patients with MGRS could have several comorbidities associated with kidney disease. Therefore, MGRS is often underdiagnosed. Additionally, identifying MGRS is challenging without the results of a kidney biopsy or monoclonal immunoglobulin study. We advocate considering MGRS as a potential diagnosis for middle-aged and older patients who present with unexplained decreased kidney function and/or proteinuria. The renal symptoms vary, and the affected segments (glomerulus, tubulointerstitium, and vasculature) of the kidney influence the clinical presentation of MGRS. Table 1 summarizes the renal presentation according to the renal lesions associated with MGRS [4, 6]. Monoclonal deposits with MGRS can affect any or all segments. Furthermore, the dominant site of monoclonal deposits determines the classification of MGRS. However, most MGRS lesions involve more than one compartment, and significant overlap exists.

**Table 1:** Renal lesions associated with monoclonal gammopathy of renal significance

Lesion (proportion of lesions, %)	Renal symptoms	Light microscopy findings	IF findings	Ultrastructural findings (deposition)
Immunoglobulin-related amyloidosis (80%)	Proteinuria, hematuria, hypertension, CKD	Congo red-positive mesangial and capillary wall deposits (dichroism + birefringence under polarized light) Vascular and tubulo-interstitial involvement (common)	AL: LC deposits, mostly λ AH: HC deposits (g1 or g4 or a) with first constant domain (CH1) deletion AHL: LC and HC deposits, mostly γ + λ or α + κ	Randomly arranged unbranched fibrils 7–14 nm in diameter (organized deposits)
MIDD (78%–100%)	Proteinuria, microscopic hematuria, hypertension, CKD	Nodular glomerulosclerosis (constant in HCDD) Thickened TBM and vascular walls	Linear deposits along the TBM and GBM and around arteriolar/arterial myocytes LCDDL: mostly κ (Vk4) HCDD: truncated HC (g1, g3, g4, or a) with CH1 deletion C3 deposits in g1 and g3 HCDD LHCDD: LC truncated HC deposits	Amorphous deposits in the TBM, GBM, mesangium, and arteriolar/arterial walls (nonorganized deposits)
PGNMID (96%)	Proteinuria, microscopic hematuria	Membranous proliferative GN, endocapillary proliferative GN, mesangial GN	Granular deposits in the mesangium and capillary wall Monotypic IgG deposits IgG3 (most common), IgG1, or IgG2 (k41) Rarely, monotypic IgM, IgA, or LC deposits C3 C1q deposits	Nonorganized granular deposits in the mesangium, subendothelial, and/or subepithelial zone (nonorganized deposit)
C3 glomerulopathy with monoclonal gammopathy (40%–90%)	Proteinuria, microscopic hematuria, hypertension, CKD	Membranous proliferative GN, endocapillary proliferative GN	Granular C3 deposits in the mesangium and capillary wall No or few Ig deposits	Sausage-shaped intramembranous and large, rounded mesangial electron-dense deposits III-defined, mesangial, intramembranous, and subendothelial electron-dense deposits in C3GN humps common in DDD and C3GN (nonorganized C3 deposit)
Monoclonal fibrillary GN (55%–98%)		Mesangial proliferative GN, mesangial expansion, amorphous eosinophil material in the capillary wall	Global mesangial and glomerular capillary wall, ill-defined deposits (IgG, C3, κ, and λ)	Randomly arranged 12-nm to 24-nm fibrils in the GBM and mesangial matrix (organized deposit)
Type I cryoglobulinemic GN (47%–52%) and type II cryoglobulinemic GN (20%)	Proteinuria, microscopic hematuria, hypertension, AKI, anuria, CKD	Membranous proliferative GN, endocapillary GN, glomerular thrombi (common)	Granular deposits in the mesangium, capillary wall (predominantly subendothelial), vascular walls, and glomerular thrombi Monotypic IgG, IgM, or IgA (κ > λ) C3, C4, and C1q deposits	Microtubules (10–90 nm), extracellular and intracellular crystals (organized deposit)
Immunotactoid GN (25%–50%)	Proteinuria, microscopic hematuria, hypertension	Mesangial GN with membranous features, membranous proliferative GN	Granular/smudgy deposits in the mesangium and capillary wall (predominantly subepithelial) Monotypic IgG deposits (IgG14, IgG24, IgG3) (k41) C3, C4, and C1q deposits	Parallel-arranged microtubules (10–60 nm) with a hollow core (organized deposit)
Cryocrystalglobulin or crystal globulin nephropathy (18%)	Proteinuria, pyuria, CKD	Crystals in all capillary lumens, glomerular epithelium, and convoluted tubules	Large crystals in the vessels and glomeruli with IgG and κ	Large intraglomerular capillary electron-dense crystal (organized deposit)
Crystal-storing histiocytosis (8%)	Fanconi syndrome	Histiocytes with crystalline inclusions in the interstitium and perirenal fat PTC atrophy and dedifferentiation	PTC LC inclusions, mostly κ	Crystals (needle-shaped) within histiocytes and occasionally in the PTC and glomerular cells (organized deposit)

*AH* Immunoglobulin heavy chain amyloidosis, *AHL* Immunoglobulin heavy chain and light chain amyloidosis, *AL* Immunoglobulin light chain amyloidosis, *CKD* Chronic kidney disease, *DDD* Dense deposit disease, *IF* Immunofluorescence, *Ig* Immunoglobulin, *GBM* Glomerular basement membrane, *GN* Glomerulonephritis, *HC* Heavy chain, *HCDD* Heavy chain deposition disease, *LC* Light chain, *LHCDD* Light chain deposition disease, *MIDD* Monoclonal immunoglobulin deposition disease, *PGNMID* Proliferative glomerulonephritis with monoclonal gammopathy, *PTC* Peritubular capillary, *TBM* Tubular basement membrane

Patients with MGRS may experience decreased kidney function, proteinuria, and microscopic hematuria. Some patients may present with proximal tubular dysfunction. Proximal tubular dysfunction can accompany Fanconi syndrome, which includes normoglycemic glycosuria, hypophosphatemia caused by hypophosphaturia, aminoaciduria, hyperuricosuria, and wasting of urinary bicarbonate [5]. Additionally, MGRS can mimic kidney diseases such as membranous nephropathy and anti-glomerular basement membrane antibody disease [30, 31]. Recurrent kidney disease after kidney transplantation could suggest masked MGRS with primary kidney disease [30]. A rapid diagnostic assessment is critical for patients with MG, and a kidney biopsy should be performed to evaluate the MGRS type and assess the severity of kidney lesions. Only 3.7% of patients with MG have experienced major hemorrhagic complications after kidney biopsy; this rate is similar to that of the control population [32]. Therefore, a kidney biopsy is considered a safe procedure for MGRS. Furthermore, the results of the kidney biopsy will reveal the cause of MG and kidney dysfunction, such as a decreased glomerular filtration rate and proteinuria. One study showed that most patients (63%) with serum and/or urine MG who underwent kidney biopsy had diseases unrelated to MG [33]. Hence, the results of the kidney biopsy are essential to diagnosing kidney lesions in patients with MG.

#### Long-term outcomes of MGRS

The long-term outcomes of MGRS remain unclear. Patients who were diagnosed early during the course of renal failure and who did not require renal replacement therapy experienced better outcomes [26]. Because the kidney is the first organ damaged by toxic monoclonal proteins, the renal response may be the primary goal of MGRS treatment [26]. Exploring genomic changes that occur with MGRS may improve our understanding of it, resulting in better treatment [26]. Recent studies have reported mortality rates of 20% to 39% during follow-up (follow-up range, 30–60 months) [24–27].

### Diagnostic tool for MGRS

Differentiating MGRS from MGs, which are not associated with kidney disease, is critical. Investigations should be performed to determine the presence of monoclonal immunoglobulins when patients present with unexplained kidney disease. This study highlights the need to maintain a low threshold for clinically suspected MGRS, particularly in middle-aged and older individuals. When MGRS is suspected, both a kidney biopsy and hematologic evaluation are essential.

#### Kidney biopsy

Clinicians must fully consider the benefits and risks of a kidney biopsy. Because appropriate treatment can improve patients' kidney outcomes with MGRS, the underdiagnosis of MGRS may lead to the need for dialysis. A kidney biopsy is recommended for patients with MG who also have unexplained kidney disease, those with CKD who exhibit an atypical clinical course, and those with MG who are younger than 50 years [4].

Biopsy specimens must be evaluated using light microscopy and immunofluorescence with light-chain, heavy-chain, and intact immunoglobulin antibodies. To confirm the monotypic nature of immunoglobulin deposits, immunofluorescence staining for immunoglobulin G subclasses should be performed. The identification of complement C1q and/or C3 proteins might suggest MGRS-related lesions such as PGNMIDs, immunotactoid glomerulonephritis, cryoglobulinemia glomerulonephritis, C3 glomerulopathy, or heavy chain and light chain deposition diseases. Electron microscopy is often necessary to identify specific MGRS lesions [4]. However, immunofluorescence may yield false-negative or false-positive results in some cases [34, 35]. Therefore, for selected cases, more sophisticated techniques, such as immunogold labeling or proteomics via laser microdissection and mass spectrometry, are required [4, 5]. Ultrastructural immunogold labeling is a sensitive technique that can help determine the histopathological diagnosis of renal lesions in patients with MGRS [35]. Mass spectrometry is a sensitive and specific tool used for diagnosing and acute typing of renal amyloidosis, including immunoglobulin heavy chain amyloidosis [36]. 

#### Monoclonal immunoglobulin testing

Serum protein electrophoresis and serum immunofixation electrophoresis (IFE) are the gold standards for diagnosing MG [37]. However, the limitations of the laboratory performing these tests could lead to inconsistent results. Urine protein electrophoresis and urine IFE are also highly sensitive; however, the results may be unclear if renal function is impaired. To compensate for these shortcomings, a free light chain assay can be used as an alternative method of diagnosing MG. The serum M-spike concentration and serum free light chain assay have diagnostic and prognostic importance for the severity and type of kidney disease [5, 6]. International guidelines recommend using a serum-free light chain assay along with serum protein electrophoresis, and IFE as part of the initial screening for MG. This approach is advised because of its increased sensitivity and the potential limitations of urinary assessments in patients with renal impairment [38, 39]. However, serum IFE and urine IFE are advised to allow for optimal sensitivity when attempting to diagnose amyloid light chain amyloidosis and light chain deposition disease [40].

#### Clonal identification

After confirming MG and monoclonal proteins using the results of the kidney biopsy, clonal identification of MG should be performed [4]. Clonal identification refers to the characterization of an underlying clonal cell population, which is crucial to developing a therapeutic approach. When dangerous clones such as the B-cell clone, LPL clone, and plasma cell clone are found, a specific diagnostic work-up including peripheral blood flow cytometry, bone marrow aspiration with flow cytometry and biopsy, computed tomography and/or positron emission tomography-computed tomography evaluations, immunoglobulin M level quantification, MYD88 mutation testing, and whole-body computed tomography should be performed [19]. Computed tomography and/or positron emission tomography can help locate focal lesions in the bone marrow [11].

#### Treatment of MGRS: hematologic and kidney-specific treatment for MGRS

Collaboration between hematologists and nephrologists is important when treating MGRS. Currently, no known specific treatment can prevent M-protein tissue deposition and improve the kidney function of patients with MGRS. However, chemotherapy that targets B-cell clones should be administered. Autologous peripheral blood stem cell transplantation is a viable option for treating B-cell clones [41]. MGRS should be monitored to prevent thrombosis and infection, which can occur with nephrotic syndrome [41]. Because chemotherapy and autologous bone marrow transplantation have been addressed elsewhere, we focused on kidney-specific treatment in this work [41]. Conservative kidney treatment comprising bicarbonate, phosphate, and vitamin D supplementation should be administered to prevent CKD-related mineral and bone disorders. Renin–angiotensin–aldosterone system inhibitors, such as angiotensin-converting enzyme inhibitors and angiotensin receptor blockers, should be considered for patients with hypertension. Patients with ESRD require dialysis, and kidney transplantation is the best treatment option [42]. However, high rates of recurrent nephropathy that were not fully eradicated before kidney transplantation have been observed among patients with MGRS [43, 44]. Although no definite evidence confirms the usefulness of MGRS treatment, a large, randomized, controlled study showed that sodium glucose cotransporter-1 inhibitors and finerenone improved both the renal outcomes and mortality rates associated with CKD [45–47]. Therefore, these factors should be considered when determining the treatment for MGRS.

#### Kidney transplantation for MGRS with ESRD

The definite role of kidney transplantation in MGRS has not been determined. Patients with MGRS are more likely to experience recurrence, even after kidney transplantation. The appropriate time for kidney transplantation is also unclear [48]. However, kidney transplantation may be a feasible treatment option for patients with MGRS and ESRD because hematologic treatment can improve the prognosis and survival rate. Patients with a positive response to rescue therapy have experienced good graft survival. Therefore, kidney transplantation is recommended for patients who have experienced good outcomes with effective rescue therapy, despite the high recurrence rate [49]. When renal function declines because of MGRS, kidney transplantation can be considered a treatment method. However, the degree of hematologic remission at the time of kidney transplantation and the recurrence rate and prognosis of the underlying disease are important indicators of graft loss. Consequently, even when kidney transplantation is required because of MGRS, close monitoring of the underlying hematologic disease is essential. Appropriate hematologic treatment before kidney transplantation is the most important factor associated with avoiding renal failure in patients with MGRS and ESRD.

## Conclusion

MGRS presents a significant challenge in clinical practice because of its varied presentation that overlaps with other conditions associated with CKD. Therefore, the importance of early detection, accurate diagnoses, and appropriate management cannot be overstated. A multidisciplinary approach and management are essential to improving the understanding of MGRS and its outcomes.

## Data Availability

No datasets were generated or analysed during the current study.
